# Planar, Twisted, or Curved Extended Tetrathiafulvalenes with Polycyclic Aromatic Hydrocarbon Cores Featuring Five‐Membered Rings

**DOI:** 10.1002/asia.202500356

**Published:** 2025-04-09

**Authors:** Viktor Bliksted Roug Pedersen, Mogens Brøndsted Nielsen

**Affiliations:** ^1^ Department of Chemistry University of Copenhagen Universitetsparken 5 Copenhagen Ø DK‐2100 Denmark

**Keywords:** Aromaticity, Conjugation, Fused‐ring systems, Polycycles, Sulfur heterocycles

## Abstract

Tetrathiafulvalene (TTF) and polycyclic aromatic hydrocarbons (PAHs) represent two distinct classes of redox‐active chromophores. By merging these two classes in so‐called extended tetrathiafulvalenes, new physico‐chemical characteristics emerge. Here in this study, we summarize the properties of such PAH‐extended TTFs for which the PAH core incorporates five‐membered carbo‐cyclic rings together with six‐, seven‐, and/or eight‐membered rings. A key structural motif of these molecules is the planar 2‐(1*H*‐inden‐1‐ylidene)‐1,3‐dithiole unit. Fusing two such units directly together or via various one‐ or two‐dimensional PAH scaffolds has during the past decade provided a large selection of planar, twisted, or curved extended TTFs. Strong associations of radical cations are in some cases obtained, corresponding to redox‐controlled self‐assembly in solution, and these associations have allowed for the ready generation of semi‐conducting salts by electrocrystallization. Some molecules exhibit remarkable multiredox behavior and can reversibly reach high cationic states, and, in some cases, also anionic states. Several structural examples also reveal how closed‐shell versus open‐shell characters of the dication states can be controlled by the nature of the PAH core, particularly by the number of Clar sextets within the core.

## Graphical abstract

Tetrathiafulvalenes (TTFs) extended by polycyclic aromatic hydrocarbons (PAHs) constitute a class of redox‐active chromophores. This review summarizes PAH‐extended TTFs for which the PAH core features five‐membered rings together with six‐, seven‐, and/or eight‐membered rings. The redox and optical properties depend on the geometrical shape of these molecules as well as the degree of PAH aromaticity of the oxidized species.

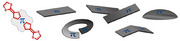

## 1 Introduction

**Scheme 1:**
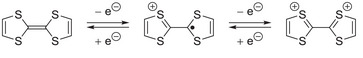
Reversible, two‐electron oxidations of tetrathiafulvalene (TTF).

Tetrathiafulvalene (TTF) is a Weitz‐type,^[^ ^1^ ^]^ redox‐active molecule that undergoes two reversible one‐electron oxidations, ultimately generating a dication containing two 6π‐aromatic 1,3‐dithiolium rings (Scheme 1).^[^ ^2^ ^]^ The optical and redox properties of TTF can be finely tuned by peripheral substituents as well as by separating the two 1,3‐dithiole rings by a π‐conjugated spacer,^[^ ^3^ ^]^ thereby furnishing so‐called extended TTFs. On account of its tunable properties, TTF has found wide applications in materials chemistry as well as in supramolecular chemistry.^[^ ^4^ ^]^

**Figure 1:**
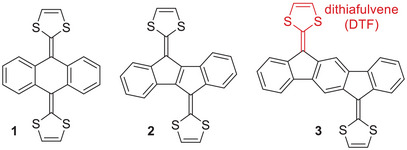
Examples of PAH‐extended TTFs.

Polycyclic aromatic hydrocarbons (PAHs) are a class of compounds that consists of fused, conjugated aromatic rings, usually five‐ or six‐membered rings, but other ring sizes can also be present.^[^ ^5^ ^]^ The acenes or polyacenes are a subgroup of the PAHs, containing only fused six‐membered rings. One of the most explored classes of extended TTFs has an oligoacene as the π‐conjugated spacer between the two dithiole rings.^[^ ^[Link]^, ^[Link]^ ^]^ Such extended TTFs have 1,4‐dithiafulvene (DTF) units located at six‐membered rings, with an exocyclic carbon–carbon double bond separating the dithiole and six‐membered rings. On account of steric interactions between the sulfur atoms of the dithiole rings and hydrogen atoms at the acene core, such extended TTFs are usually nonplanar. They often undergo a two‐electron oxidation that is accompanied by a significant geometrical change in the molecule. For example, compound **1** (and its derivatives) shown in Figure 1 changes geometry upon two‐electron oxidation from a butterfly‐like structure to a planar anthracene core with two 1,3‐dithiolium appendages.^[^ ^3d^ ^]^ Similarly, large conformational changes were observed upon two‐electron oxidation of oligoacene‐extended TTFs for which two‐dimensional and planar anthanthrene or tetraceno[2,1,12,11‐*opqra*]tetracene PAH cores are generated.^[^ ^6^ ^]^

**Figure 2:**
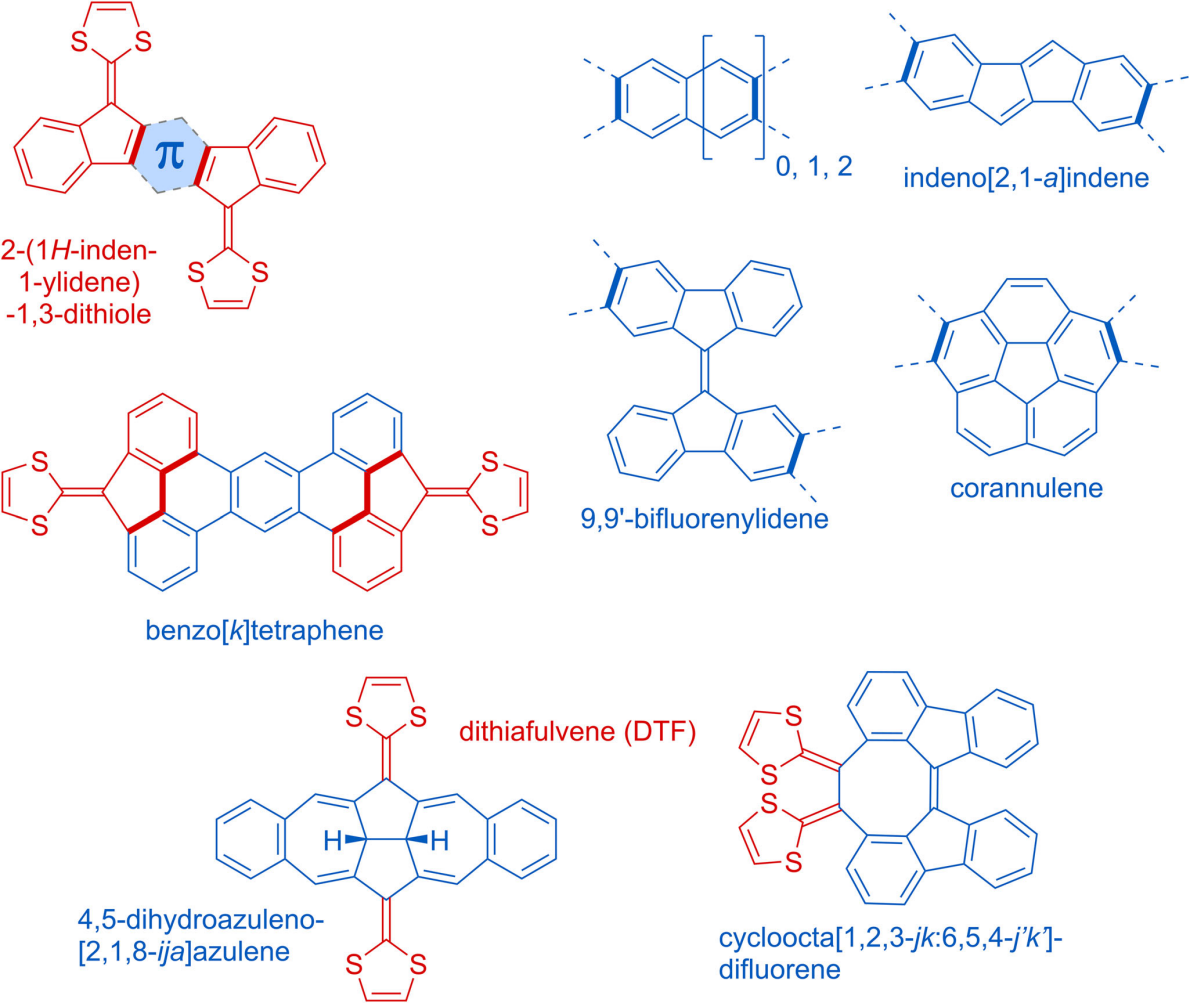
Overview of PAH‐extended TTFs covered in this review. For the spacers shown at the top, right, the two thick bonds highlighted correspond to the bonds shared with the two thick bonds highlighted of the 2‐(1*H*‐inden‐1‐ylidene)‐1,3‐dithiole (in red) units at the top, left. For the two structures shown at the bottom, the names of the cores correspond to the cores that are generated upon two‐electron oxidation.

While acene‐extended TTFs have been the most explored of the PAH‐extended TTFs, the last decade has seen an increased focus on PAH‐extended TTFs with odd‐membered rings as part of the PAH core. These include structures where DTF units are placed at five‐membered rings, such as the general structures **2** and **3** shown in Figure 1.^[^ ^3^, ^7^, ^8^ ^]^ As described below, this often allows for co‐planarity of the PAH and dithiole rings. Here, we will focus on these and other molecules featuring five‐membered carbo‐cycles as part of the PAHs, the structures of which have been generalized in Figure 2. A common structural motif is the planar 2‐(1*H*‐inden‐1‐ylidene)‐1,3‐dithiole unit. Fusing two such motifs generates compound **2**,^[^ ^7^ ^]^ while fusing them to a benzene ring can generate the indeno[1,2‐*b*]fluorene‐extended TTF **3** (or other isomers).^[^ ^8^ ^]^ Figure 2 shows a variety of other one‐ or two‐dimensional, planar, or nonplanar motifs that have been inserted between two 2‐(1*H*‐inden‐1‐ylidene)‐1,3‐dithiole units. Moreover, DTF units have been placed at 4,5‐dihydroazuleno[2,1,8‐*ija*]azulene and cycloocta[1,2,3‐*jk*:6,5,4‐*j'k’*]difluorene cores. The redox properties are strongly dependent on the nature of the PAH core as we shall systematically cover below.

**Scheme 2:**
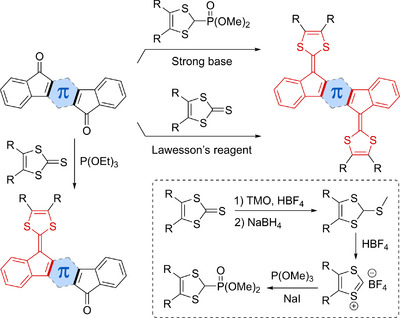
General synthetic routes to extended DTFs/TTFs and the conversion of a 1,3‐dithiol‐2‐thione into a phosphonate. TMO = trimethyl orthoformate. Lawesson's reagent = 2,4‐bis(4‐methoxyphenyl)‐1,3,2,4‐dithiadiphosphetane‐2,4‐dithione.

Synthetically, most compounds were prepared by Horner–Wadsworth–Emmons olefination reactions by treating the corresponding PAH‐diketone with a deprotonated dimethyl (1,3‐dithiol‐2‐yl)phosphonate (Scheme 2).^[^ ^3e^ ^]^ Other synthetic approaches involve treating the diketone with a 1,3‐dithiol‐2‐thione by the action of Lawesson's reagent (useful as well for generating the bifluorenylidene motif from carbonyl precursors).^[^ ^9^ ^]^ Triethylphosphite‐mediated olefinations between the diketone and a 1,3‐dithiol‐2‐thione usually only resulted in the introduction of one DTF unit at the PAH core,^[^ ^8^ ^]^ which is convenient for constructing unsymmetrical compounds. The phosphonate is as illustrated in Scheme 2 prepared in a few steps from the 1,3‐dithiol‐2‐thione, taking advantage of a recently disclosed protocol for the initial methylation step using trimethyl orthoformate instead of hitherto used dimethylsulfate.^[^ ^10^ ^]^

## 2 PAH‐Extended TTFs Featuring Five‐Membered Rings

### 2.1 Indenofluorene Regioisomers and Expanded Structures—Planarity versus NonPlanarity

**Figure 3:**
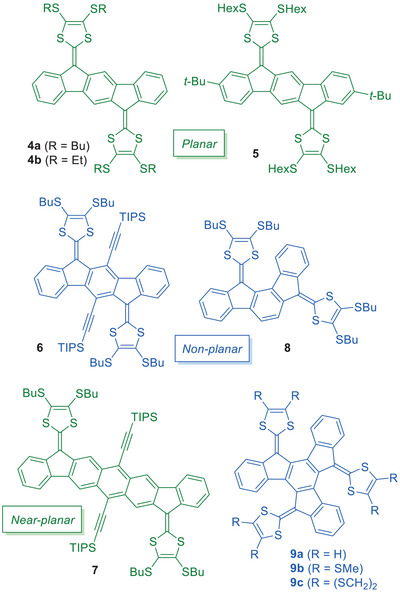
Regioisomers of indenofluorene‐extended TTFs and expanded versions. TIPS = triisopropylsilyl.

Several regioisomers of indenofluorene‐extended TTFs and related structures have been reported to date. Compounds **4a** and **4b**, as shown in Figure 3, contain the indeno[1,2‐*b*]fluorene core and were introduced by Haley, Hammerich, Nielsen, and co‐workers.^[^ ^8^ ^]^ Cyclic voltammetry studies of **4a** showed (at a concentration of 1 mM) two reversible one‐electron oxidations at 0.24 and 0.42 V versus Fc/Fc^+^ in CH_2_Cl_2_. The stepwise oxidations contrast the behavior of the smaller compound **2** with a central pentalene core (Figure 1) that instead underwent a single two‐electron oxidation (at 0.42 V versus SCE in MeCN + 0.1 M Et_4_NBF_4_; SCE = standard calomel electrode).^[^ ^7^ ^]^ The first oxidation wave of **4a** was observed to be broad and concentration dependent. This behavior was ascribed to intermolecular associations between two neutral molecules, a neutral and a radical cation (mixed valence, MV, and complex) and two radical cations (π‐dimer). Association constants for these complexes were found to be very high, 1 × 10^4^ M^−1^ (MV complex) and 4 × 10^3^ M^−1^ (π‐dimer). For comparison, the MV dimerization constant of the parent TTF has been determined to only 6 M^−1^ in CH_2_Cl_2_ at 22 °C.^[^ ^11^ ^]^ The π‐dimer of two TTF radical cations was only detected at very low temperature (−70 to −90 °C) in CH_2_Cl_2_, but the association constant could be determined to 0.6 M^−1^ in acetone at 22 °C.^[^ ^11^, ^12^ ^]^ The strong associations of indenofluorene‐extended TTFs arise from the planarity of the molecule (as confirmed by single crystal X‐ray crystallography of the neutral and monocationic species of **4b**, vide infra), which allows these favorable intermolecular interactions to occur. Further oxidation to the dication results in disruption of the self‐assembled structures. Associations in solution were supported by spectroelectrochemical studies; thus, oxidation of **4a** resulted in characteristic near‐infrared absorptions at 1450, 1650, and 1996 nm (in CH_2_Cl_2_ + Bu_4_NPF_6_), which were assigned to the π‐dimer **[4a•4a]^2+^**, MV dimer **[4a•4a]^•+^**, and monomer radical cation **4a^•+^**, respectively. Further oxidation to the dication **4a^2+^** caused the disappearance of these absorptions and the emergence of a new characteristic absorption at 905 nm. Upon electrocrystallization of **4b**, semi‐conducting salts comprised of layered radical cations were obtained.^[^ ^8^ ^]^

Associations between molecules were diminished by the introduction of bulky *tert*‐butyl substituents at the periphery of the indeno[1,2‐*b*]fluorene core.^[^ ^13^ ^]^ Thus, compound **5** (Figure 3) only showed slight broadening of the first oxidation peak at high concentrations. This compound exhibited oxidations at 0.27 and 0.39 V versus Fc/Fc^+^ in CH_2_Cl_2_. However, linking together two such units by flexible bridges resulted in intramolecular associations of the oxidized species.^[^ ^13^ ^]^ Moreover, intramolecular associations were observed for dimers (devoid of the *tert*‐butyl substituents) containing aryl‐alkynyl^[^ ^14^ ^]^ or indigo bridges.^[^ ^15^ ^]^

Distortion of the indeno[1,2‐*b*]fluorene‐extended TTF from planarity was accomplished by attachment of bulky triisopropylsilylethynyl substituents at the central benzene ring.^[^ ^16^ ^]^ The resulting compound **6** (Figure 3) underwent a single two‐electron oxidation at 0.31 V versus Fc/Fc^+^. Expanding the core to an anthracene unit (compound **7**; Figure 3), while maintaining the bulky alkynyl substituents at the central benzene ring, restored a near‐planar system according to calculations, and this compound showed two reversible one‐electron oxidations at 0.28 and 0.48 V versus Fc/Fc^+^.^[^ ^16^ ^]^

The indeno[1,2‐*a*]fluorene‐extended TTF regioisomer **8** shown in Figure 3 is not planar, as per the calculated structure, due to the steric congestion between the DTF unit and the central π‐system.^[^ ^16^ ^]^ The disruption of planarity is observed as significant increases in the two reversible one‐electron oxidation potentials, now at 0.34 and 0.53 V versus Fc/Fc^+^. In addition to the lack of planarity, the two DTF units are located in a *meta*‐configuration relative to the central benzene ring, disrupting the conjugation along the molecule, while the two rings are connected in a *para*‐configuration in **1**. Introducing a third 2‐(1*H*‐inden‐1‐ylidene)‐1,3‐dithiole unit has furnished compounds **9a**‐**c** (Figure 3), reported by Martín and co‐workers.^[^ ^17^ ^]^ These truxene‐extended TTFs are concave and showed more complex redox properties. Thus, cyclic voltammograms of compounds **9b** and **9c** showed two broad waves. Interestingly, the concave shape of these molecules allows them to bind fullerenes. Thus, **9a** formed 1:1 complexes with C_60_ and C_70_ with association constants of 1.2 × 10^3^ M^−1^ and 8.0 × 10^3^ M^−1^, respectively, in CDCl_3_/CS_2_ 1:1.^[^ ^17^ ^]^ Moreover, **9a** was found to form supramolecular complexes with π‐extended corannulene derivatives, in which intermolecular photoinduced electron transfer processes were found to occur according to photophysical studies.^[^ ^18^ ^]^

**Figure 4:**
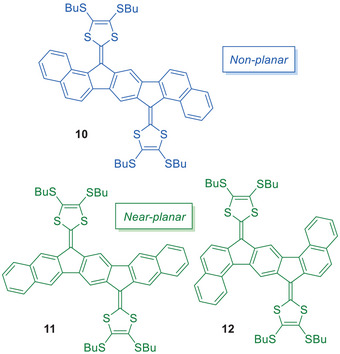
Elongated, benzo‐fused derivatives of indenofluorene‐extended TTFs.

**Figure 5:**
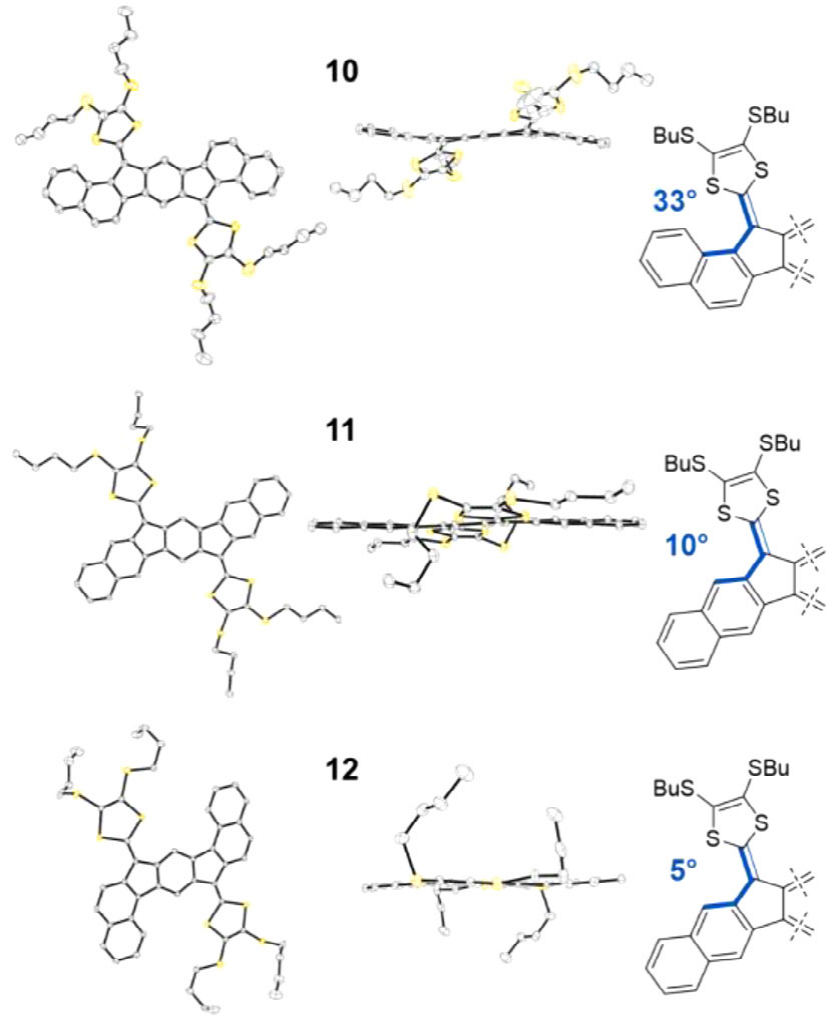
Molecular structures of **10**–**12**, obtained from X‐ray crystallographic analysis (hydrogen atoms have been omitted). Torsion angles are indicated to the right. Figure reproduced (modified version) with permission from Ref. [16].

Annulation of a benzene ring on both sides of the indeno[1,2‐*b*]fluorene‐extended TTF yields the three elongated regioisomers **10**, **11**, and **12** as shown in Figure 4.^[^ ^16^ ^]^ While compounds **11** and **12** are almost planar, compound **10** is significantly twisted out of planarity with a torsion angle between each DTF unit and its neighboring naphthalene unit of 33°. Torsional angles of **10**–**12**, obtained from X‐ray crystallographic analysis, are summarized in Figure 5. Compound **11** showed a broad first oxidation event corresponding to a two‐electron process affected by associations in solution (CH_2_Cl_2_). Compound **12** also showed associations in solution which led to two broad oxidation events associated with the formation of the radical cation and complexes at 0.11 and 0.35 V versus Fc/Fc^+^, followed by oxidation to the dication at 0.49 V versus Fc/Fc^+^. In stark contrast, the nonplanar compound **10** showed a single two‐electron oxidation event at low potential, 0.28 V versus Fc/Fc^+^. The low oxidation potential can be explained by relief of the steric congestion of the dithiole ring upon oxidation, as the exocyclic fulvene double bond obtains single‐bond character. The benzannulations strongly affect the optical properties as well. The longest‐wavelength absorption maxima are *λ*_max_ = 504 nm (**10**), 478 nm, weak shoulder around 525 nm (**11**; with SHex instead of SBu peripheral substituents), and 502 nm (**12**). For comparison, compound **4a** has a longest‐wavelength absorption at *λ*_max_ = 475 nm.

### 2.2 Closed‐ or Open‐Shell Dications as a Function of the PAH Core

**Figure 6:**
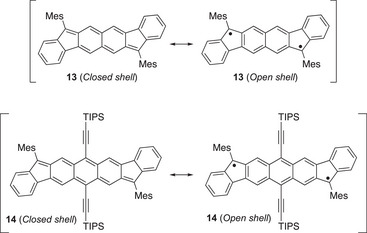
Closed and open‐shell structures of substituted fluoreno[3,2‐b]fluorene and diindeno[1,2‐b:1′,2′‐i]anthracene compounds. Mes = mesityl.

**Figure 7:**
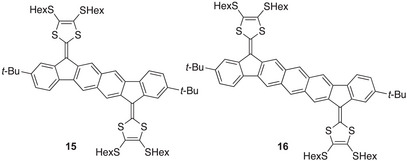
Extended TTFs containing central naphthalene and anthracene units.

The dications of the extended TTFs consist of two aromatic 1,3‐dithiolium rings and a PAH core that is devoid of exocyclic fulvene bonds. One key question is whether the generated PAH core is a closed‐ or open‐shell structure. The dication of **4a** has been found to be ESR‐silent, and it seems to be best described by a closed‐shell, quinoid structure.^[^ ^8^ ^]^ This description corresponds to that of a neutral alkynyl‐substituted indenofluorene[1,2‐*b*]fluorene as reported by Haley and co‐workers.^[^ ^19^ ^]^ However, upon expanding the central core to naphthalene or anthracene moieties, corresponding to the fluoreno[3,2‐*b*]fluorene and diindeno[1,2‐*b*:1′,2′‐*i*]anthracene derivatives **13** and **14** shown in Figure 6, some degree of singlet open‐shell character was obtained (with a thermally accessible triplet excited state of **14**).^[^ ^20^, ^21^ ^]^ The corresponding extended TTFs **15** and **16** (Figure 7) were therefore prepared and studied.^[^ ^22^ ^]^ Here naphthalene and anthracene have been formally fused to two 2‐(1*H*‐inden‐1‐ylidene)‐1,3‐dithiole units.

**Figure 8:**
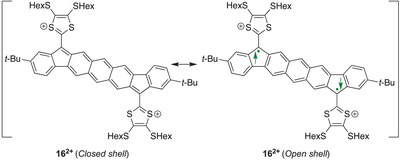
Closed‐ versus open‐shell singlet structures of the dication of 1‐dimensional PAH‐extended TTF.

The dications **15^2+^** and **16^2+^** were found to be ESR‐silent, and they thus feature a singlet ground state.^[^ ^22^ ^]^ Broken‐symmetry calculations on **16^2+^** (with SHex substituents truncated to SMe) revealed, however, that the closed‐ and open‐shell singlets shown in Figure 8 are essentially isoenergetic. Some diradicaloid character of this dication seems thus reasonable, which is in line with the properties observed for the parent PAH core **14**.^[^ ^21^ ^]^ The cyclic voltammogram of **16** showed (in contrast to that of **15**) a very broad wave for the second oxidation, and it is speculated whether this could relate to reversible radical dimerizations. Furthermore, spectroelectrochemical studies revealed a longest‐wavelength absorption maximum for this dication (1290 nm) at particularly low energy.

**Figure 9:**
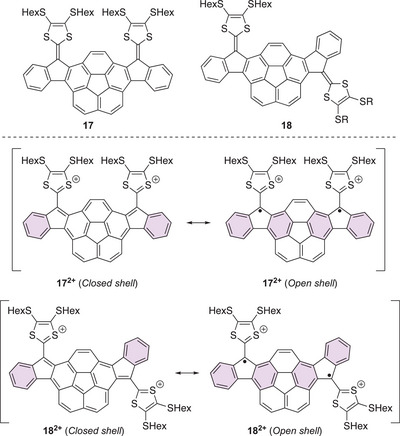
Corannulene‐extended TTFs and closed‐ versus open‐shell structures of their corresponding dications. Clar sextets are highlighted as violet‐colored filled rings.

Together with Jasti and co‐workers,^[^ ^23^ ^]^ we introduced corannulene as a spacer between two 2‐(1*H*‐inden‐1‐ylidene)‐1,3‐dithioles, furnishing extended TTFs **17** and **18** (Figure 9). The dications of these bowl‐shaped molecules seemed to have some diradicaloid character as they were found to be ESR‐active. They were reversibly generated under cyclic voltammetry conditions, but when generated in bulk, they were found to undergo reactions (as observed by a decrease in the characteristic absorption at 950 nm over time), signaling reactive radical behavior. As described for the related neutral and mesityl‐substituted structures (with no exocyclic double bonds),^[^ ^24^ ^]^ diradicaloid character of these compounds is promoted by an increased number of Clar sextets: four Clar sextets can be drawn for the open‐shell structures and only two for the closed‐shell structures. Chemical oxidation of **17** and **18** with “Magic Blue” (tris(4‐bromophenyl)ammoniumyl hexachloroantimonate) resulted in new absorptions around 950 nm and a weak band in the region 1000–2000 nm. Further oxidation to the dications only intensified these absorptions, which correlates with a diradicaloid species and minimum communication across the corannulene central motif.

**Figure 10:**
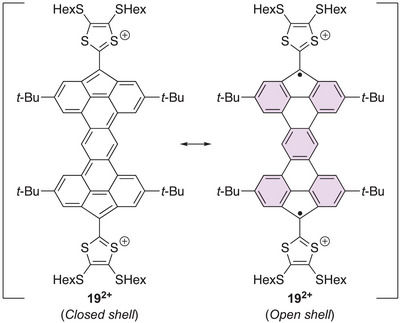
Closed‐ versus open‐shell structures of fluoreno[4,5‐klm]indeno[2,1,7‐def]tetraphene‐extended TTF dications. Clar sextets are highlighted as violet‐colored filled rings.

An even further increase in the number of Clar sextets for the open‐shell structure was found for the dication of the bis‐fluorene‐fused compound **19** shown in Figure 10, which we recently reported together with Morin and co‐workers.^[^ ^25^ ^]^ For the fluoreno[4,5‐*klm*]indeno[2,1,7‐*def*]tetraphene core of the dication, the closed‐shell structure has zero Clar sextets, while the open‐shell structure has as many as five. This dication showed ESR activity, and calculations revealed the open‐shell (triplet) state to be 10.4 kcal mol^−1^ more stable than the corresponding closed‐shell singlet state. In line with this result, a substituted derivative of the neutral PAH core (with no exocyclic double bonds) was found to exhibit an open‐shell triplet ground state.^[^ ^25^ ^]^

### 2.3 Extended TTFs with Acceptor Properties—Multi‐Redox Systems

**Figure 11:**
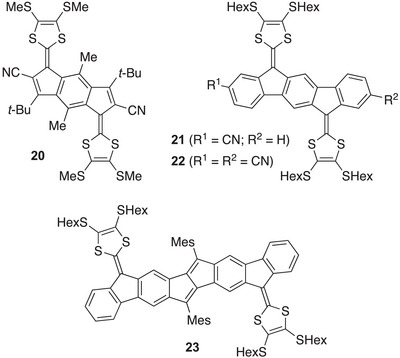
One‐dimensional PAH‐extended TTFs with both donor and acceptor properties.

**Scheme 3:**
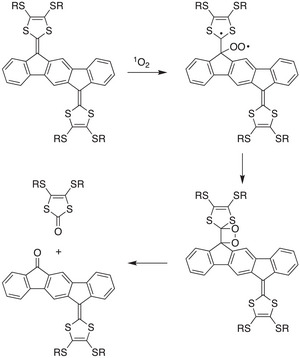
General reaction scheme for the reaction of indenofluorene‐extended TTFs with singlet oxygen, supported by computations (R = Me) and isolation of mixture containing the two end‐products (R = Hex).

In general, the larger the π‐system, the more redox states can be achieved. Multi‐redox behavior involving both reversible oxidations and reductions requires, however, specific structural modifications of the extended TTFs. For example, indacene‐ and indenofluorene‐extended TTFs do not act as electron acceptors under cyclic voltammetry conditions (within a reasonable threshold of − 2.1 V versus Fc/Fc^+^), but the introduction of cyano substituents at the core provides molecules **20**,^[^ ^26^ ^]^ **21**,^[^ ^27^ ^]^ and **22** ^[^ ^27^ ^]^ (Figure 11), which can be both oxidized and reduced. Thus, compounds **21** and **22** are reduced at −2.04 and −1.84 V versus Fc/Fc^+^, respectively, in CH_2_Cl_2_. They undergo first oxidations at 0.36 and 0.42 V versus Fc/Fc^+^, respectively, and hence the cyano substituents render them weaker donors than **4a**. These two compounds were found to exhibit a reduced reactivity towards singlet oxygen by reducing the nucleophilicity of the exocyclic fulvene bond (cleaving it into two carbonyl compounds, Scheme 3), which is of interest in the design of stable chromophores for potential device applications.^[^ ^27^ ^]^

Another strategy for achieving acceptor properties (along with donor properties) is to expand the one‐dimensional PAH core. Replacing the central benzene ring of the indenofluorene core with a naphthalene (**15**) or an anthracene (**16**) is not sufficient,^[^ ^22^ ^]^ but elongating the core to a pentaleno[1,2‐*b*:4,5‐*b'*]difluorene, acceptor properties are obtained without the need for adding acceptor substituents.^[^ ^28^ ^]^ Thus, compound **23** was found to undergo a reduction at −1.74 V versus Fc/Fc^+^ in CH_2_Cl_2_.

**Figure 12:**
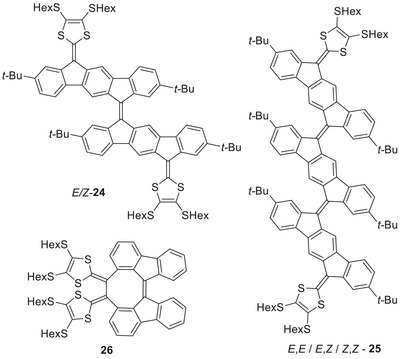
Two‐dimensional PAH‐extended TTFs with both donor and acceptor properties.

Expanding the central PAH to a two‐dimensional core has also provided acceptor properties, as exemplified by the compounds shown in Figure 12. Compounds **24** and **25** with extended bifluorenylidene spacers are particularly strong acceptors, undergoing first reductions at −1.52 and −1.32 V versus Fc/Fc^+^, respectively.^[^ ^9^ ^]^ These nonplanar compounds exhibit redshifted longest‐wavelength absorptions at 605 nm (**24**) and 675 nm (**25**) relative to that of the indenofluorene‐extended TTF **4a** (475 nm). Studies revealed that **24** exhibits solvatochromism, which indicates charge‐transfer character of the longest‐wavelength absorption. In accordance with this result, computations revealed that the HOMO extends along the molecule from one DTF to the other, while the LUMO is mainly located at the central spacer of the molecule.

Incorporating the bifluorenylidene into a cycloocta[1,2,3‐*jk*:6,5,4‐*j“k”*]difluorene core, as in the extended TTF **26** (Figure 12), also furnishes good electron acceptor properties;^[^ ^29^ ^]^ this compound undergoes a first reduction at −1.55 V versus Fc/Fc^+^. Corannulene‐extended TTFs **17** and **18** (Figure 9) are also electron acceptors, albeit less strong, being reduced irreversibly at −1.99 and −1.98 V versus Fc/Fc^+^, respectively.^[^ ^23^ ^]^

**Figure 13:**
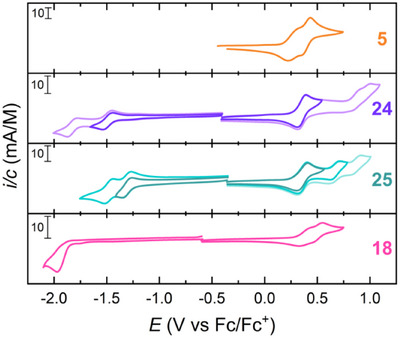
Cyclic voltammograms of selected donor‐acceptor multi‐redox systems **18**, **24**, and **25** in comparison to that of donor **5**.

Figure 13 shows the cyclic voltammograms of the donor–acceptor multi‐redox systems **18**, **24**, and **25** in comparison to that of the indenofluorene‐extended TTF donor **5**. The large systems **24** and **25** even showed a second reversible reduction at low potential. They both underwent two‐electron oxidations (at 0.34 and 0.35 V versus Fc/Fc^+^, respectively) and further oxidations at higher potential. Thus, compound **25** underwent two subsequent oxidations to reach the tetracationic structure, allowing it to reach six different redox states almost fully reversibly; −2, −1, 0, +2, +3, +4.^[^ ^9^ ^]^

### 2.4 PAH Cores Incorporating Seven‐Membered Rings

Introducing odd‐membered rings to the central π‐system can change the character of the dicationic species. The conjugation of the π‐system plays a large role in whether the systems are open‐shell or closed‐shell structures, as discussed above with Clar sextets. Moreover, generation of a Hückel‐aromatic system upon oxidation can counter‐balance Coulomb repulsion in the dication, reducing the separation between first and second oxidation potentials or even resulting in a two‐electron oxidation as observed for the acene‐extended TTF **1** for which an aromatic anthracene core is generated.^[^ ^3d^ ^]^

**Figure 14:**
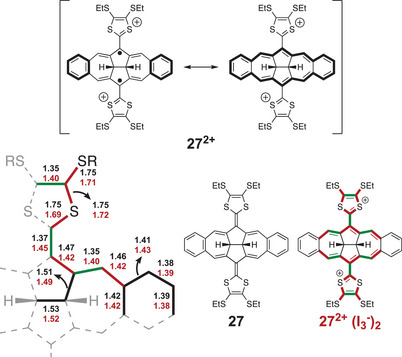
Neutral and dication structures of extended TTF with a PAH core that includes both five‐ and seven‐membered rings. Bond lengths of the neutral compound (black numbers) and the dication (red numbers) are shown in the lower left corner. Red bonds indicate shortening and green bonds indicate elongation of the corresponding bonds upon oxidation to the dication. Figure reproduced (with modifications) with permission from Ref. [30].

The introduction of 4,5‐dihydroazuleno[2,1,8‐*ija*]azulene as a central nonbenzenoid PAH core, annulated with benzene rings, yields a system, compound **27** (Figure 14), for which the dication is obtained in a two‐electron oxidation at low potential (0.17 V versus Fc/Fc^+^).^[^ ^30^ ^]^ The formation of an aromatic central core was supported by computational and experimental data. A diatropic ring current observed in the anisotropy of the induced current density (ACID) plot, high negative nucleus independent chemical shift (NICS) values, and the electron density of delocalized bonds (EDDB) all support the formation of a Hückel‐aromatic central π‐system in the dicationic species. Oxidation of **27** with I_2_ yielded single crystals of the dicationic species, suitable for 3D electron diffraction experiments. Analysis of the crystal structure showed that significant structural changes occur upon oxidation, including planarization of the 22π‐system and significant bond length changes. The changes in bond lengths between the neutral and dicationic species are shown in Figure 14; the exocyclic fulvene bond is significantly elongated for the dicationic species. The localized single and double bonds of the neutral species are shortened and elongated, respectively, for the dication towards bond length equivalization, which serves as experimental evidence for the formation of an aromatic, nonbenzenoid system.^[^ ^30^, ^31^ ^]^ The dication of **27** could be further oxidized; thus, a reversible one‐electron oxidation was observed at 0.69 V and an irreversible oxidation at 0.93 V versus Fc/Fc^+^.

**Figure 15:**
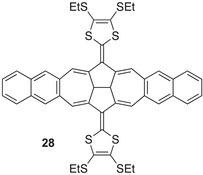
Extended TTF with dihydroazulene–azulene core expanded with naphthalene units at each end.

Expanding the core further has significant consequences for the redox properties. Thus, the naphtho‐fused compound **28** (Figure 15) shows two reversible one‐electron oxidations at +0.18 and +0.29 V versus Fc/Fc^+^,^[^ ^30^ ^]^ while the dication of **27** was generated in a two‐electron oxidation. Both compounds exhibited additional oxidations at higher potentials.

### 2.5 Structures of Mono‐ and Dicationic Species

**Figure 16:**
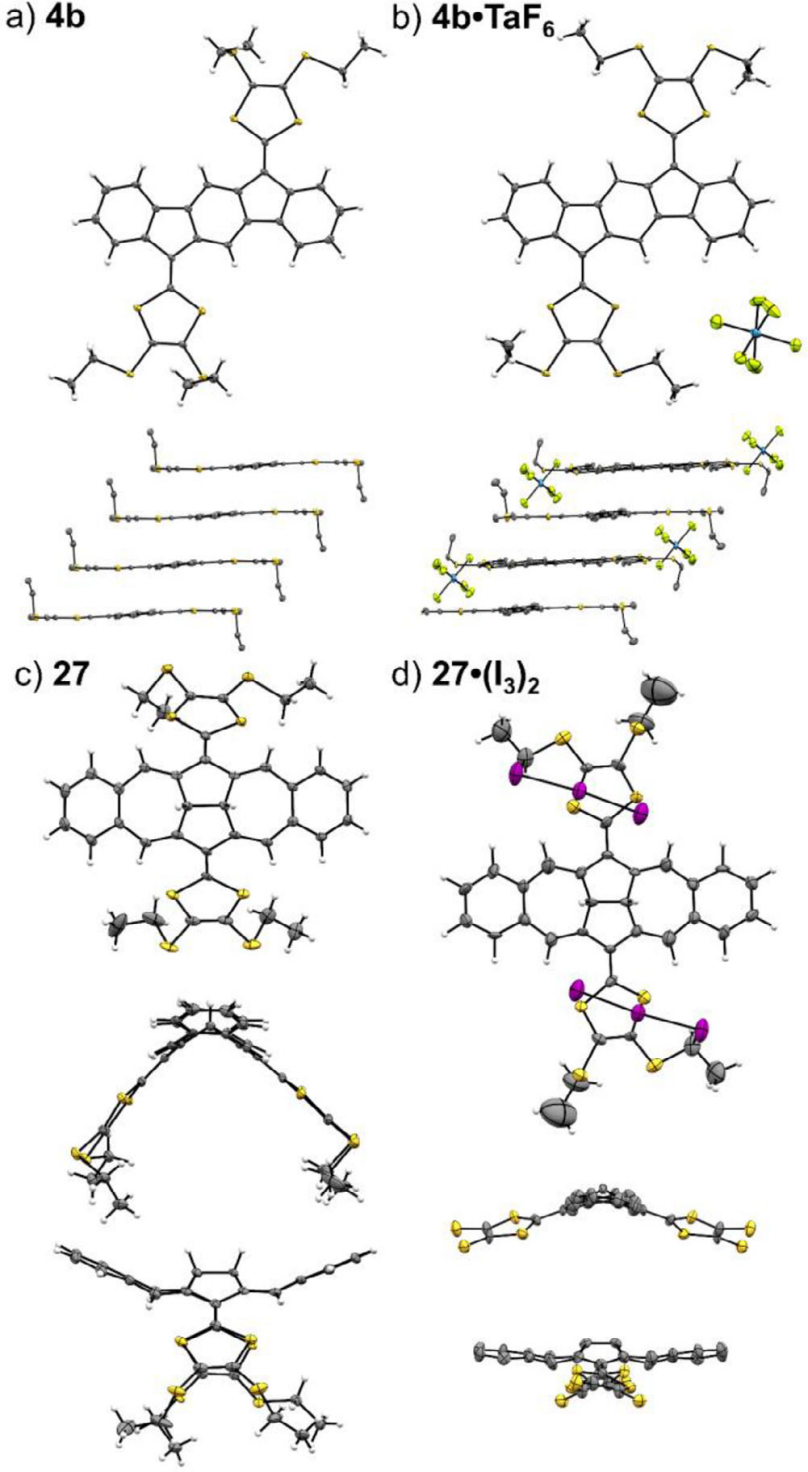
Molecular structures and crystal packings of neutral and cationic extended TTFs. a) Neutral **4b**; b) Radical cation salt of **4b**; c) Neutral **27**; d) Dication salt of **27**. Hydrogen atoms and ethyl chains have been omitted for clarity in some parts of the figure. Part of figure reproduced (with modifications) with permission from Ref. [30].

Several crystal structures of cationic extended TTFs have been reported to date. While TTF itself can obtain cationic character in the characteristic charge transfer salts for TTF or acene‐extended TTFs,^[^ ^3^, ^32^ ^]^ no such complexes have, to our knowledge, been reported to date of extended TTFs with the DTF motifs placed on five‐membered rings. The structures of cationic species have, however, been obtained through electrocrystallization experiments or by direct chemical oxidation.^[^ ^8^, ^30^ ^]^ Three salts of **4b** with different counter anions (PF_6_^−^, TaF_6_^−^, and BF_4_^−^) were prepared by electrocrystallization.^[^ ^8^ ^]^ The structures of the salts were elucidated by single‐crystal X‐ray crystallography. The monocation was formed for the PF_6_^−^ and TaF_6_^−^ salts, while a 1:1 mixture of mono‐ and dications was formed with the BF_4_^−^ counter ion. Bond lengths of the exocyclic fulvene bonds in these structures are listed in Table 1 in comparison to those in neutral **4b**. Figure 16 shows molecular structures and crystal packings of **4b** together with the **4b•**TaF_6_ salt. Notably, both the neutral and cationic species are fully planar.

**Table 1:** Average bond lengths of exocyclic fulvene bonds in neutral and cationic extended TTFs.

Compound	Length (Å)	Compound	Length (Å)
**4b**	1.363(3)	**27**	1.374(3)
**4b•**TaF_6_	1.388(21)	**27•**(I_3_)_2_	1.453(15)
**4b•**PF_6_	1.388(11)		
**4b•**(BF_4_)_1.5_	1.390(10)		

We recently reported the crystal structure of the dication of the dihydroazulenoazulene‐extended TTF **27** generated upon oxidation with iodine, furnishing triiodide as the counter anion.^[^ ^30^ ^]^ The structure was obtained by 3D electron diffraction experiments, and it revealed a significant increase in the bond length of the exocyclic fulvene bond from 1.37 (**27**) to 1.45 Å (**27^2+^**). This change in bond length is much larger than that observed for the three salts of **4a** (see Table 1). This agrees with the fact that the salts of **4a** contain the radical cation, which retains partly double bond character of the exocyclic fulvene bonds. As mentioned above, the molecular geometry changes significantly when proceeding from neutral **27** to its dication; thus, the saddle‐like structure is somewhat flattened upon oxidation (Figure 16).

## 3 Summary and Outlook

In summary, extended TTFs with PAH cores featuring five‐membered rings can be obtained in many geometries: planar, twisted, and curved. The structural modifications have strong consequences for redox as well as optical properties of neutral and oxidized species.

While indeno[1,2‐*a*]fluorene‐extended TTFs are nonplanar, indeno[1,2‐*b*]fluorene regioisomers are planar, which allows for redox‐controlled assembly of molecules as mixed valence and π‐dimer complexes with characteristic near‐infrared absorptions. Distortions from planarity of this indenofluorene regioisomer can nevertheless be promoted by bulky substituents at the central ring of the PAH core or by benzo‐fusion at a specific sites at the ends of the PAH.

The balance between diradicaloid versus quinoid character of the dications was found to depend strongly on the number of Clar sextets; by enhancing this number, more diradicaloid character can be engineered. Diradicaloid character of various extended TTF dications were in general found to correlate well with the properties of related, neutral PAH cores with no exocyclic double bonds (often substituted with mesityl groups). Further development of such motifs with redox‐controlled generation of diradicaloid character is particularly interesting in the context of spintronics and molecular electronics devices. Thus, diradicaloid structures can result in magnetic‐field controlled Kondo effects in molecular electronics junctions^[^ ^33^ ^]^ or a conductance of nanowires that increases with length (“anti‐Ohmic wires”).^[^ ^34^ ^]^

Suitable modifications of the PAH cores can provide acceptor character of the molecules in addition to donor character; for example, pentaleno[1,2‐*b*:4,5‐*b'*]difluorene, bifluorenylidene, and cycloocta[1,2,3‐*jk*:6,5,4‐*j'k'*]difluorene cores provide donor–acceptor multi‐redox systems.

Finally, it has been shown how gain of large‐perimeter Hückel aromaticity in nonbenzenoid dihydroazuleno‐azulene structures can be used to tune redox properties. These molecules were found to undergo significant geometrical changes upon oxidation.

Table 2 summarizes redox potentials and longest‐wavelength absorption maxima for most of the extended TTFs discussed in this review. Only oxidations up to the dications are listed, but most of the compounds can take higher oxidation states, albeit in some cases irreversibly. We have here focused on all‐carbon PAH cores, but several structures are also known with PAHs containing heteroatoms, such as structures incorporating fused thiophene units.^[^ ^16^, ^25^, ^30^, ^35^ ^]^ Moreover, pyrrole units have been fused to the dithiole rings in structures based on the indenofluorene core.^[^ ^36^ ^]^ Rather large two‐dimensional PAH cores have been introduced in several of the structures discussed in this review, but it would be interesting to target even larger systems in future work.

**Table 2:** Summary of first oxidation potentials (generation of mono‐ and dications^a)^ and first reduction potential^b)^ as well as longest‐wavelength absorption maxima.^c)^

Compound	*E*_ox_^1^/V	*E*_ox_^2^/V	*E*_red_^1^/V^d)^	*λ*_max_/nm
**4a** (1 mM)	0.24 (broad)	0.42		475
**4b** (0.2 mM)	0.31	0.43		473
**5**	0.27	0.39		472
**6**	0.31 (2e)			492
**7**	0.28	0.48		562
**8**	0.34	0.53		431
**10**	0.28 (2e)			504
**11**	0.30 (2e) (broad)			478^e^ ^)^
**12**	0.11/0.35 (broad)	0.49		502
**15**	0.24	0.43		471
**16**	0.16	0.37 (broad)		489
**17**	0.41	0.48	−1.99	469
**18**	0.38	0.49	−1.98	489
**19**	0.32	0.38		424^e^ ^)^
**21**	0.36 (broad)	0.49	−2.04	489
**22**	0.43	0.55	−1.84	504
**23**	0.35	0.50	−1.74	568
**24**	0.34 (2e)		−1.51	605
**25**	0.35 (2e)		−1.32	675
**26**	0.19	0.33	−1.55	599 (tail)
**27**	0.17 (2e)			493
**28**	0.18	0.29		441

^a)^ Additional oxidations are in general observed.

^b)^ Solvent: CH_2_Cl_2_; counter electrolyte: Bu_4_NPF_6_. Potentials versus Fc/Fc^+^.

^c)^ Solvent: CH_2_Cl_2_ if not otherwise stated.

^d)^ Only reductions occurring at less negative potential than −2.1 V are considered, and only the first reduction is listed. Derivative of **11** with peripheral SHex instead of SBu substituents.

^e)^ Solvent: CHCl_3_.

## Conflict of Interests

The authors declare no conflict of interest.
